# Supplementary material for: Mapping the evolution of fertility support policies in China: A content and instrumental analysis
Source: PLoS One. 2025 Oct 9;20(10):e0332137. doi: 10.1371/journal.pone.0332137 (PMC12510515; doi:10.1371/journal.pone.0332137)
Supplement: S1 Appendix — (ZIP) [file pone.0332137.s001.zip › S1 Appendix. 226 original policy documents/27-国家卫生计生委、中央宣传部、中央文明办等关于“十三五”期间深入推进婚育新风进万家活动的意见(FBM-CLI-16-277622).doc]

国家卫生计生委、中央宣传部、中央文明办等关于“十三五”期间深入推进婚育新风进万家活动的意见

**发布部门：** [国家卫生和计划生育委员会(已撤销)](https://www.pkulaw.com/law/chl?Aggs.IssueDepartment=60319) [中共中央宣传部](https://www.pkulaw.com/law/chl?Aggs.IssueDepartment=a04) [中央精神文明建设指导委员会办公室](https://www.pkulaw.com/law/chl?Aggs.IssueDepartment=a27) [机构沿革](javascript:void(0))

**发文字号：**国卫宣传发〔2016〕42号

**发布日期：**2016.07.29

**实施日期：**2016.07.29

**时效性：** [现行有效](https://www.pkulaw.com/law/chl?Aggs.TimelinessDic=01)

**效力级别：** [党内法规制度](https://www.pkulaw.com/law/chl?Aggs.EffectivenessDic=XR12)

**法规类别：** [人口计划管理](https://www.pkulaw.com/law/chl?Aggs.Category=09701)

国家卫生计生委、中央宣传部、中央文明办、教育部、民政部、文化部、新闻出版广电总局、全国总工会、共青团中央、全国妇联、中国计生协会关于“十三五”期间深入推进婚育新风进万家活动的意见
（国卫宣传发〔2016〕42号）

各省、自治区、直辖市卫生计生委、党委宣传部、精神文明办、教育厅（委、局）、民政厅（局）、文化厅（局）、新闻出版广电局、工会、团委、妇联、计生协，新疆生产建设兵团人口计生委、党委宣传部（广电局、出版局、精神文明办）、教育局、民政局、文化厅局、工会、团委、妇联、计生协：
　　全国婚育新风进万家活动自1998年开展以来，已形成了国家倡导、社会动员、群众参与的工作格局，对普及计划生育和生殖健康知识、转变群众婚育观念、传播新型婚育文化和稳定适度生育水平发挥了重要作用。
　　当前，我国人口总量增长势头明显减弱，劳动年龄人口和育龄妇女开始减少，老龄化程度不断加深；群众生育观念发生重大转变，少生优生已成为社会生育观念的主流；家庭规模小型化，养老抚幼功能弱化；我国仍处于城镇化快速发展阶段，人口流动迁移保持活跃；以互联网为代表的传播手段迅速发展，育龄群众信息接收渠道发生转变。党的十八届五中全会提出，推进健康中国建设，坚持计划生育的基本国策，完善人口发展战略，全面实施一对夫妇可生育两个孩子政策，积极开展应对人口老龄化行动，注重家庭发展。为“十三五”期间（2016-2020年）深入推进婚育新风进万家活动，提出如下意见：
　　**一、**指导思想
　　以党的十八大和十八届二中、三中、四中、五中全会和习近平总书记系列重要讲话精神为指导，以深入贯彻落实中共中央、国务院《[关于实施全面两孩政策改革完善计划生育服务管理的决定](https://www.pkulaw.com/chl/8564756bae805261bdfb.html)》为中心，以坚持计划生育基本国策、实施全面两孩政策、改革完善计划生育服务管理为主线，大力培育和践行社会主义核心价值观，创新宣传方式方法，发挥先行先导作用，建设适应新时期人口形势、促进人的健康和全面发展、体现中华传统美德和时代精神的新型婚育文化。

　　**二、**活动目标
　　面向基层、面向群众、面向家庭，以培育“婚育文明、性别平等；计划生育、优生优育；生活健康、家庭幸福”的新型婚育文化为重点，以倡导文明婚育、传播健康知识、弘扬家庭美德为主要任务，设定年度主题，动员全社会力量，通过宣传倡导、优质服务和政策推动，增强全社会的人口国情、计划生育基本国策意识，推进婚育文明建设。到“十三五”期末，80%以上地级市（州）开展婚育新风进万家活动，为全面建成小康社会，实现“中国梦”创造良好的人口环境。

　　**三、**基本原则
　　（一）争取各级党委、政府的统一领导，省、地市、县、乡、村分级科学制订方案，统筹规划目标，合理安排活动。
　　（二）以群众需求为导向，以人的全面发展为目标，不断满足群众对新时期计划生育服务的新期待，努力创新宣传思维、宣传内容和宣传手段。
　　（三）调动和发挥各级基层组织在联系群众、服务家庭方面的独特优势，以家庭为单位开展创建活动，关注家庭需求，着眼家庭发展，服务家庭成员。
　　（四）发挥各相关部门的职能作用，加强对活动的指导协调，形成工作合力。动员广大人民群众广泛参与，强化群众的主体地位。
　　（五）针对不同地区的人口形势和面临的婚育风俗主要问题，不同人群、不同家庭的实际特点和需求，开展有针对性的精准宣传活动。重点加大对边远贫困地区的工作力度，加强对弱势人群的关怀和扶助，讲求实际效果。确定年度主题，围绕年度主题策划重点宣传活动。

　　**四、**主要任务
　　（一）宣传依法按政策生育，建设新型婚育文明。广泛宣传我国人口国情以及计划生育基本国策，全面准确解读全面两孩政策，加强人口与计划生育法律法规宣传，及时公开计划生育相关服务信息，引导广大群众负责任、有计划地安排生育。落实并宣传生育登记服务制度，方便群众办事，切实使这一制度成为新的“民心工程”。传播符合社会主义核心价值观的婚姻观念，维护平等、文明、和谐的婚姻关系，倡导婚事简办，反对包办婚姻、违法早婚、大操大办和借婚姻索取财物。营造夫妻平等氛围，促进夫妻共同承担家庭责任。积极倡导保障妇女合法权益，不断提高社会性别平等意识，促进出生人口性别结构趋向自然平衡。
　　（二）推广妇幼健康优质服务，提高妇幼健康水平。深化开展关爱女孩行动。大力宣传卫生计生惠民政策，推广母子健康手册，以科学备孕、孕产期保健、安全分娩、新生儿安全为重点，制订专项健康教育计划，结合母亲节、儿童节等时间节点，对育龄人群开展有针对性的健康教育。引导孕产妇合理选择助产服务机构，动员高龄孕妇到二级以上医院、妇幼保健院接受孕产期保健服务和分娩。倡导公共场所和工作场所结合需求设置母乳喂养室。加强青少年性与生殖健康教育，减少非意愿妊娠。加强更年期生理心理教育，提供更年期生殖保健服务。
　　（三）倡导健康生活方式，促进家庭幸福。广泛传播健康科学知识和理念，提升全民健康意识和健康素养，培养文明健康的生活方式，提高群众的自我保健能力和健康水平。广泛传播健康老龄化和积极老龄化的理念和实践，提升老年人幸福感。关怀计划生育家庭，妥善解决计划生育特殊家庭的生活照料、养老保障、大病治疗和精神慰藉等问题。引导家庭成员树立社会主义核心价值观，弘扬中华民族尊老爱幼等传统美德，强化家庭养老抚幼功能与亲情氛围，树立良好家风家规。

　　**五、**工作要求
　　（一）各级党委政府和相关部门要进一步加强组织领导，将婚育新风进万家活动纳入重要议事日程、纳入计划生育目标管理责任制，健全组织机构，制订发展计划。要进一步完善经费投入保障机制，为活动顺利开展提供有力保障。
　　（二）建立健全部门协调机制，各司其职，形成合力。定期召开部门联席会议，制定具体措施。积极动员工会、共青团、妇联、计生协等群众团体以及各类非政府组织成为推动活动的重要力量。
　　（三）持续深入开展婚育新风进万家活动宣传，充分运用传统和新兴媒体，通过报刊、广播、电视、互联网、手机短信等，广泛传播婚育新风尚。鼓励和引导婚育家庭题材文艺作品创作，发挥新家庭文化屋效用，促进“送文化”与群众需求有效对接，加大政府对面向基层文艺产品和服务的购买力度。改进宣传品开发制作，坚守传统宣传阵地，多使用育龄群众喜闻乐见的动漫、游戏、流行歌曲、微视频等形式，使宣传更加深入人心。利用重大节日、纪念日、活动日等宣传节点组织形式多样的社会宣传活动，吸引广大人民群众和志愿者广泛参与。
　　附件：部门职责

国家卫生计生委
中央宣传部
中央文明办
教育部
民政部
文化部
新闻出版广电总局
全国总工会
共青团中央
全国妇联
中国计生协会
2016年7月29日

　　附件

　　部门职责

　　党委宣传部门：把婚育新风进万家活动纳入本部门总体工作规划和年度工作计划，列入文化、科技、卫生“三下乡”活动安排，协调报刊、广播、电视和互联网等媒体，做好相关的宣传报道工作。
　　精神文明建设部门：把婚育新风进万家活动纳入精神文明创建活动，结合文明家庭创建，推动家庭婚育文化建设。
　　教育部门：学校教育与家庭教育、社会教育相结合，利用相关课程和主题活动，引导青少年了解我国的人口国情，普及青春期健康教育知识，提高青少年自我保健能力，提高青少年的家庭责任感，促进青少年的身心健康。
　　民政部门：为开展婚前保健、计划生育、优生优育等宣传教育提供便利。在民生工程、乡镇服务型政府建设、街道服务管理创新和社区建设中，开展家庭婚育文化建设，发展社会福利，提供社会保障和社区服务，优先照顾和扶持实行计划生育的家庭，做好老年人服务和养老保障工作。
　　文化部门：引导、鼓励创作以婚育新风为主题的群众文艺作品，充分发挥文艺团体、公益性文化单位以及公共数字文化工程的重要作用，大力宣传和普及家庭婚育文化知识。
　　卫生计生部门：制定婚育新风进万家活动第五阶段工作方案，细化年度工作方案并组织实施，指导家庭婚育文化建设，落实活动项目，组织单位开展工作。开展生殖健康和计划生育咨询服务工作，开展计划生育、优生优育、妇幼保健和性别平等的宣传教育、咨询与技术服务。
　　新闻出版广电部门：鼓励制作出版以婚育新风为主题的电影、电视剧、专题片、科普片、图书、音像制品、电子出版物等文化产品和宣传品，并配合卫生计生部门做好对婚育新风进万家活动的重点宣传，防止负面炒作。加大新家庭文化屋的普及和推广力度。
　　工会：把婚育新风进万家活动纳入工会组织的宣传教育工作，发挥工会报刊、网站和工人文化宫、职工书屋等工会文化设施的作用，在青年职工、特别是农民工中积极倡导婚育新风。
　　共青团：号召青年志愿者积极参与到婚育新风进万家活动中，引导广大青年适龄结婚，婚事简办；适龄生育，按政策生育；计划生育，优生优育。
　　妇联：倡导妇女适龄婚育，反对早婚早育。结合基层妇女工作，组织妇女积极参与婚育新风进万家活动，与“双学双比”、“巾帼建功”、“五好文明家庭创建”和“寻找最美家庭”等活动有机结合。
　　计生协会：动员协会会员和群众广泛参与婚育新风进万家活动。与“5·29会员活动日”、计生基层群众自治、生育关怀行动、少数民族生殖健康促进、幸福家庭创建活动等工作相结合，围绕计划生育、优生优育、生殖健康、帮扶计生家庭等方面，开展多种形式的宣传服务活动，倡导科学文明生育观念，提高群众生殖健康意识，依法维护计生家庭合法权益，促进家庭和谐幸福。

© 北大法宝：（[www.pkulaw.com](https://www.pkulaw.com/)) 专业提供法律信息、法学知识和法律软件领域各类解决方案。北大法宝为您提供丰富的参考资料，正式引用法规条文时请与标准文本核对。
欢迎查看所有[产品和服务](https://www.pkulaw.com/helps/2.html)。法宝快讯：[如何快速找到您需要的检索结果？](https://www.pkulaw.com/helps/70.html)[法宝](https://www.pkulaw.com/helps/69.html)V6有何新特色？

扫描二维码阅读原文

**原文链接：**<https://www.pkulaw.com/chl/274be5c9c2a93c71bdfb.html>
